# Secondary Leukemia Associated with the Anti-Cancer Agent, Etoposide, a Topoisomerase II Inhibitor

**DOI:** 10.3390/ijerph9072444

**Published:** 2012-07-10

**Authors:** Sachiko Ezoe

**Affiliations:** Hematology and Oncology, Osaka University Graduate School of Medicine, 2-2 Yamada-oka Suita, Osaka 565-0871, Japan; Email: sezoe@bldon.med.osaka-u.ac.jp; Tel.: +81-6-6879-3871; Fax: +81-6-6879-3879

**Keywords:** etoposide, secondary leukemia, MLL, treatment

## Abstract

Etoposide is an anticancer agent, which is successfully and extensively used in treatments for various types of cancers in children and adults. However, due to the increases in survival and overall cure rate of cancer patients, interest has arisen on the potential risk of this agent for therapy-related secondary leukemia. Topoisomerase II inhibitors, including etoposide and teniposide, frequently cause rearrangements involving the *mixed lineage leukemia* (*MLL*) gene on chromosome 11q23, which is associated with secondary leukemia. The prognosis is extremely poor for leukemias associated with rearrangements in the *MLL* gene, including etoposide-related secondary leukemias. It is of great importance to gain precise knowledge of the clinical aspects of these diseases and the mechanism underlying the leukemogenesis induced by this agent to ensure correct assessments of current and future therapy strategies. Here, I will review current knowledge regarding the clinical aspects of etoposide-related secondary leukemia, some probable mechanisms, and strategies for treating etoposide-induced leukemia.

## 1. Introduction

Drugs that induce secondary leukemias comprise alkylating agents, like cyclophosphamide, topoisomerase II inhibitors, including epipodophylotoxins (etoposide, teniposide), and anthracyclinic agents, like doxorubicin.

Etoposide (VP-16, 4′-dimethylepipodophylloxin-9-[4,6-O-ethylidene-beta-D-glucopyranoside]) is an agent that targets DNA topoisomerase II. It has been widely used for the treatment of many types of cancers in children and adults [1]. However, etoposide has been associated with an increased risk of secondary leukemia. In 1987, Ratain *et al.* first propounded the relationship between etoposide and secondary leukemia when it was used as a therapy for advanced non-small-cell lung cancer [2]. Subsequently, several investigators reported secondary leukemias, particularly acute myelogenous leukemia (AML), associated with translocations of the *mixed lineage leukemia* (*MLL*) gene at human chromosomal band 11q23 when etoposide was used to treat lung cancer, non-Hodgkin lymphoma, neuroblastoma, acute lymphoid leukemia (ALL), Wilms tumor, and rhabdomyosarcoma, [3,4,5]. The mechanism that leads to MLL-specific rearrangement is largely unknown. However, recent studies have reported some speculative mechanisms based on *in vitro* experiments. In this paper, the findings from previous reports that described the clinical aspects, some probable mechanisms, and treatments for etoposide-related secondary leukemia will be summarized.

## 2. Aspects of AML after Etoposide Treatments

Alkylating agents cause secondary AML characterized by antecedent myelodysplasia, and the typical latency period for the development of secondary leukemia is about 5–7 years. Often (60–90% of cases), complete or partial deletion of chromosome 5 or 7 occurs, and patients exhibit the M1 or M2 phenotype (French-American-British (FAB) classification; Table 1) [6,7]. The risk of causing secondary myelodysplastic syndrome (MDS) and leukemia associated with alkylating agents mainly depends on the cumulative dose of alkylating agents. Predisposing factors for the alkylating agent-associated secondary leukemias are germline *NF-1* [8] and *p53* gene mutations [9] and a null *GSTT1* genotype [10], and Ras mutations are frequently accompanied with those leukemias [11,12]. On the other hand, epipodophyllotoxins and other DNA topoisomerase II inhibitors are linked to leukemias that bear *MLL* gene translocations at chromosome bands 11q23, t(8;21), t(3;21), inv(16), t(8;16), t(15;17), or t(9;22) [9]. Generally the leukemias following epipodophyllotoxins-treatments are likely to occur within 3 years after treatments, and the mean latency period from drug administration to the onset of secondary leukemia is about 2 years. Between 2 and 12% of patients that receive epipodophyllotoxin develop secondary AML. Most cases of epipodophyllotoxin-induced leukemia exhibit FAB M4 or M5 morphology, but observations have also included other FAB AML subtypes, MDS, ALL, and chronic myelogenous leukemia (CML). The prognosis of alkylating-agent-induced secondary leukemia appears to be worse than that for spontaneously occurring leukemias, and the prognosis of etoposide-related secondary leukemia is extremely poor. Pui *et al.* [13] reported that, out of 21 patients with etoposide-related secondary-leukemia, only two could maintain complete remission. Furthermore, Sandler *et al.* [14] reported that, even with therapy, including bone marrow transplantation, the 2-year disease free survival rate was 17.6% for children’s epipodophyllotoxin-induced secondary leukemia.

However, single-agent therapy may be rather uncommon in cancer treatments. Epipodophyllotoxins are generally used as part of alkylating agents- or cisplatin-based protocls in treatments of solid tumors [15,16], and are given with cytarabine in leukemia treatment [17]. *In vitro* study, combinations of etoposide with an alkylating agent or sisplatin produce synergistic cytocidal effects on leukemic cell. And In the Intergroup Rhabdomyosarcoma Study III, the risk of secondary AML was significantly higher for children receiving cyclophosphamide and etoposide compared to those receiving cyclophosphamide without etoposide [18]. Thus in assessments of the risk of secondary leukemia following etoposide therapy, we must consider the combination of other cytotoxic agents.

**Table 1:** Characteristics of secondary leukemia following epipodophyllotoxin or alkylating agent treatment.

	Epipodophyllotoxin	Alkylating agent
Typical interval following therapy	2–3 years	5–7 years
FAB classification	M4/M5	M1/M2
Karyotype abnormalities	11q3 (MLL gene rearrangement)	(–5)/del(5q), (–7)/del(7q)
Preceding myelodysplasia	rare	frequent

## 3. Schedule and Dose of the Drug

The risk of secondary AML appears to be dependent on both treatment schedule and dose. Typically, epipodophyllotoxin-induced AML occurs after multiple doses administered in brief intravenous infusions. The 4- to 5-year cumulative risk of the complication has ranged from 0% to 18.4% in patients treated with cumulative doses ranging from 5,200 mg/m^2^ to 19,200 mg/m^2^ [19]. Currently, low-dose, chronically administered regimens are more commonly given to patients with refractory or relapsed cancers; however, it is difficult to assess the risk of drug-induced AML associated with this regimen due to the short survival period. Caution must be exercised when speculating that leukemogenesis might occur less frequently with prolonged oral dosing; nevertheless, almost all cases of epipodophyllotoxin-induced AML have been reported after administration of short intravenous infusions [20]. Moreover, it is clear that factors other than cumulative dosage may predispose patients to developing this devastating adverse effect, like the administration schedule of epipodophyllotoxin (etoposide or teniposide) and other drugs that may be given concurrently.

Chen *et al.* [21] suggested, based on their *in vitro* site-specific DNA recombination analysis, that prolonged exposure to low-dose etoposide regimens did not produce less cytotoxicity than high-dose, short-term regimens; however, low-dose regimens induced less site specific, nonhomologous DNA recombination, which suggested that more prolonged dosage schedules might achieve adequate effects with lower risk of leukemogenesis.

Etoposide is used in many treatment regimens for various tumors, including several high-dose chemotherapy regimens. Pedersen-Bjergaard reported that the risk of developing secondary leukemia increased 336-fold with etoposide doses above 2.0 g/m^2^ compared to doses of 2.0 g/m^2^ or less [22]. Thereafter, several studies have investigated the possibility of a correlation between the risk of secondary AML and the administered cumulative dose of etoposide [20]. Based on some reports, the probability of secondary leukemia within 5 years after chemotherapy for testicular cancer was about 0.6% with cumulative etoposide doses up to 2.0 g/m^2^, but 3.4% in regimens with etoposide doses above 2.0 g/m^2^. This suggested that high-dose etoposide, especially higher than 2.0 g/m^2^ of cumulative dose, was associated with increased risk for developing secondary leukemia [23,24].

On the other hand, Pui *et al.* [13] reported that secondary AML was diagnosed in 21 out of 734 patients with newly diagnosed AML that had entered complete remission and were randomly assigned to receive continuation (maintenance) treatment; secondary AML was the initial adverse event in 17 of those patients. In that study, the probability that AML developed within six years was 3.8% for any patient in continuous complete remission; however, in patients that received teniposide or etoposide in weekly or twice-weekly doses for prolonged periods, the risk was approximately 12 times higher than that of patients treated according to other schedules. Nevertheless, they could not determine reliably whether the cumulative dose of epipodophyllotoxins played a major role in the development of AML. The recognition that etoposide is dependent on the cell cycle and proliferation [25] has led to the development of more prolonged dosage schedules. Doses given over a period of several days have shown improved antitumor effects compared to equivalent total doses given as a single, short intravenous infusion [26]. Sugita *et al.* reported that a B-8801 protocol for B-cell lymphoma, which contained a cumulative etoposide dose of 10,000 mg/m^2^, was not related to the occurrence of secondary leukemia; they suggested that the dose method was more important than the cumulative dose for leukemogenesis [27].

Furthermore, additional factors influence the risk of etoposide-related secondary leukemia, like concomitant radiotherapy or high doses of other agents, including platinum agents. In patients with refractory germ cell tumors, a treatment option of high-dose chemotherapy, including high-dose etoposide, was not related to a higher incidence of leukemogenesis than standard-dose chemotherapy regimens. However, a combination of high-dose platinum agents, like cisplatin or carboplatin, may promote etoposide-induced leukemogenesis [28].

Based on these data, some regimens have been developed that reduce the risk of secondary leukemia, including reduced doses of etoposide. For example, when reduced dose chemotherapy was given to patients with intermediate-risk neuroblastoma, the 3-year overall survival was greater than 90% and the cumulative incidence of secondary leukemia was 0.7% [29]. In the Children’s Oncology Group therapeutic trial (INT-0091), Ewing sarcoma was treated with Regimen A, which included doxorubicin (375 mg/m^2^) and cyclophosphamide (20.4 g/m^2^), and Regimen B, which included doxorubicin (375 mg/m^2^), cyclophosphamide (9.6 g/m^2^), ifosfamide (90 g/m^2^), and etoposide (5 g/m^2^); this regimen achieved a low rate (<1%) of secondary leukemia [30].

## 4. The Mechanism of Induction of Secondary Leukemia by Etoposide

Most chromosomal translocation breakpoints in 11q23 are located within an 8.3-kb breakpoint cluster region (BCR), which extends from exon 7 to exon 13 of the *MLL* gene [31,32]. However, it is largely unknown how etoposide induces 11q23 chromosome translocations in this region. The *MLL* gene spans 100 kb, encodes a 430-kDa protein homologous to the Drosophila *trithorax* gene, and has important functions in embryogenesis and hematopoiesis [33,34]. MLL fusion proteins induce high expression of *MEIS1* and *HOX* genes through an epigenetic function, and thus, they inhibit differentiation and induce immortalization of immature hematopoietic cells [35]. Multiple lines of evidence have shown that etoposide-associated genomic alterations coincide with topoisomerase II cleavage activity, particularly within the *MLL* locus [36,37]. Etoposide addition to *in vitro* topoisomerase II cleavage assays enhanced DNA-topoisomerase II cleavage complexes within DNA substrates that contained *MLL* and partner gene sequences near translocation breakpoint sites identified in therapy-related leukemias [38,39]. Similarly, exposure of hematopoietic progenitor cells to etoposide led to detectable DNA-topoisomerase II cleavage complexes within the BCR, and some rearrangements were detectable after 16–24 h of continuous etoposide exposure in many hematopoietic cell lines [37,40] Sensitivity of the *MLL* locus may be a general consequence of chromatin architecture. MLL is an actively transcribed, open chromatin region throughout early hematopoietic development, and it contains a scaffold-associating region/matrix-associating region (SAR/MAR) that may be a site-specific target for topoisomerase II activity [41,42,43].

DNA damage activates the DNA damage response and repair pathways. Clinical data suggest that the MLL 11q23 locus is particularly susceptible to etoposide-mediated cleavage and subsequent illegitimate repair events that anneal heterologous chromosomes. Alternatively, etoposide may promote cleavage and illegitimate repair along all chromosomes with relatively equal frequency, but the specific oncogenic fusions with MLL lead to a growth advantage, and ultimately tumorigenesis, in a critical hematopoietic stem-cell subpopulation. Libura *et al.* [44] detected illegitimate repair events, including MLL tandem duplications and translocations (44%), in primary hematopoietic CD34+ cells treated with 20–50 mM etoposide *ex vivo*. Blanco *et al.* [45] reported that etoposide also induced MLL translocation in mouse ES cells. Bueno *et al.* [46] was the first study to use human embryonic stem cells (hESCs) as a model for testing the effects of etoposide in human embryonic development. Continuous exposure to etoposide induced MLL breaks and primed hESCs to acquire other major karyotype abnormalities. This linked embryonic genotoxic exposure to genomic instability. Recently, Vlasova *et al.* [47] reported that etoposide, which contains a hindered ring phenol, was oxidatively activated by myeloperoxidase (MPO) to a free radical species. These etoposide radicals resulted in redox cycling, which may play a role in enhanced etoposide genotoxicity. MPO expressed in CD34+ myeloid progenitor cells may make these cells particularly sensitive to the leukemogenic action of etoposide.

More recently, Cowell *et al.* explained the topoisomerase IIb dependent mechanism through which etoposide induces translocations [48]. For the effective repair of the etoposide-induced DNA double-strand breaks (DSBs) a specific machinery, Non-Homologous End-Joining (NHEJ), is required [49,50,51]. The hypersensitive sites for DNase I are also present in the BCRs of other transcription genes including AF9, AF4, RUNX1, ETO, and RARA. Cowell showed, using RNA-FISH analysis, that topoisomerase IIb is corecruited with components of the NHEJ machinery, which induces dynamic close proximity of MLL and t-AML-associated translocation partner genes, such as AF9 and AF4. This recruitment of genes to the shared machinery may contribute to the aberrant repairs in secondary AMLs.

The ataxia-telangiectasia mutation (ATM) was also reported to be involved in chromosome translocations involving 11q23. The ATM protein regulates the DNA damage response to DNA-double-strand breaks through its kinase activity [52]. Sun *et al.* [53] showed that ATM deficiency resulted in excessive binding of the DNA recombination protein, RAD51, at a translocation breakpoint hotspot on the 11q23 chromosome after etoposide exposure. This suggested that ATM modulated the loading of recombinational repair proteins onto translocational hotspots to avoid inappropriate recombination that could lead to chromosome translocation. Daurinol is a novel catalytic inhibitor of human topoisomerase IIa; its structure is quite similar to etoposide. However, unlike etoposide, it induces S-phase arrest through the ATM/Chk/Cdc25A pathway, without DNA cleavage or severe DNA damage [54]. This agent may represent an advantageous alternative to etoposide, because it may provide lower risk for developing secondary leukemias.

## 5. Treatments for Etoposide-Induced Leukemia

As pointed out above, patients with etoposide-related secondary leukemias with *MLL*-rearrangements have a significantly worse prognosis compared to patients with leukemia cells that possess a normal *MLL*. Currently, intense strategies are being developed, including stem-cell transplantation, to treat *MLL*-rearranged acute leukemias, but the outcome has remained poor. More recently, the National Cancer Institute/National Institute of Health Developmental Therapeutics Program (NCI/NIH) proposed testing an approved set of drugs designed to combat *MLL*-rearranged pediatric leukemia cell lines [55]. Results from that study showed that 42 of the 89 agents tested had measurable cytotoxicity against leukemia cells. Among those, 12 agents were effective against all five *MLL*-rearranged cell lines; these included cladribine, dactinomycin, daunorubicin, docetazel, etoposide, gemcitabine, mitomycin C, mitoxantrone, teniposide, topotecan, triethylnemelamine, and vinblastine. These data are extremely informative, and may facilitate further clinical trials for testing new treatment strategies for leukemia with *MLL* abnormalities.

## 6. Conclusions

In conclusion, it is necessary to gain more exact knowledge of secondary leukemia, identify the optimal therapy administration method, including the application schedule and dosage, and perform long-term follow-up studies to advance the prevention and treatment of secondary leukemias.
